# Predictors for Anxiety and Stress in Long COVID: A Study in the Brazilian Population

**DOI:** 10.3390/ijerph22020258

**Published:** 2025-02-12

**Authors:** Daniel de Macêdo Rocha, Andrey Oeiras Pedroso, Laelson Rochelle Milanês Sousa, Elucir Gir, Renata Karina Reis

**Affiliations:** 1 Department of Nursing, Federal University of Mato Grosso do Sul, Coxim 79400-000, Brazil; 2 Ribeirão Preto College of Nursing, University of São Paulo, Ribeirão Preto 14040-902, Brazil; apedroso@usp.br (A.O.P.); egir@eerp.usp.br (E.G.); rkreis@eerp.usp.br (R.K.R.); 3 Nursing Course, State University of Maranhão, Coroatá 65665-000, Brazil; laelsonmilanes@gmail.com

**Keywords:** post-acute COVID-19 syndrome, anxiety, stress, risk factors

## Abstract

Anxiety and stress are major challenges for public health and represent significant symptoms in long COVID. Despite the repercussions on quality of life and mental health, their impacts have not been systematically consolidated in the Brazilian population. Our objective was to analyze the indicators and predictors of anxiety and perceived stress in people who have experienced long COVID in different regional contexts in Brazil. This cross-sectional survey was carried out in the five regions of Brazil and included 4239 adult individuals who had at least one diagnosis of COVID-19. Participants responded to questions on the Depression, Anxiety, and Stress Scale (DASS-21). The GAMLSS class of regression models estimated the predictors associated with the outcomes investigated. The results showed a predominance of participants with a single diagnosis of COVID-19 (65.4%), mild clinical conditions (89.5%), and high adherence to immunization strategies (98.4%). Overall, 48.5% of participants had residual symptoms that started between 4 and 12 weeks after the acute phase of COVID-19 infection. Positive screening for anxiety and perceived stress was associated with female gender, diagnosis of chronic diseases, presence of physical symptoms, moderate or severe clinical condition in the acute phase of the infection, and the need for hospitalization. Through this study, we confirmed that anxiety and stress, developed or exacerbated during the post-COVID-19 phase, represent significant challenges in the Brazilian population. Sociodemographic, clinical, and care conditions were predictors of the outcomes assessed. Knowing these repercussions can allow for personalizing mental health care and help structure evidence-based public policies.

## 1. Introduction

Long COVID is a complex syndrome and represents a public health challenge in low-, middle-, and high-income countries [1]. It is one of the most frequent long-term effects observed in COVID-19 survivors, regardless of the severity of the acute phase of the infection or the need for hospitalization [2]. Its epidemiological, economic, social, and health impacts are documented in the literature and indicate severe compromises in functional capacity, health indicators, and the population’s quality of life [3]. Globally, surveillance initiatives to estimate the burden of the disease at a population level are not very significant [4].

Data from the World Health Organization (WHO) show that in 2022, long COVID syndrome affected the quality of life of at least 65 million individuals [5]. In the United States, a survey carried out by the Centers for Disease Control and Prevention (CDC) indicated that 6.9% of adults and 1.3% of children experienced self-reported long COVID [6,7]. The reported prevalence in the UK was 2.9% in 2023 [8]. Other estimates show that the burden in the general population is converging towards a global prevalence of 7% [7,8,9]. The literature also reports a variation in the incidence of long COVID of 10 to 30% for non-hospitalized cases, 70% for hospitalized patients, and up to 12% for vaccinated cases. In Brazil, the syndrome has also been reported at all stages of infection when in the acute phase [10].

The manifestations of the post-COVID syndrome can involve a wide clinical and laboratory spectrum, as well as variation in terms of symptoms, intensity, and duration. In this population, neuropsychiatric conditions are common and comprise debilitating features of mental health [11]. Different studies have reported a substantial increase in emotional distress among COVID-19 survivors [12,13].

In addition to cognitive dysfunction, memory deficits, declining attention, and impaired language and learning skills, psychiatric changes from COVID-19 may include symptoms of anxiety and stress [11]. Recent studies indicate the development and intensification of anxiety and stress in long COVID 4 to 12 weeks after the acute phase of COVID-19 infection and highlight the lack of association with alternative diagnosis [2,14]. Anxiety and stress symptoms are some of the main causes of emotional distress and disability in the general population [15]. A systematic review highlighted these outcomes as the most frequent psychiatric symptoms in long COVID and demonstrated their association with absenteeism, loss of productivity, decline in functionality, and increased risk of suicide [16].

A national survey carried out in the UK estimated that after the acute phase of COVID-19, 43.1% of patients showed changes in mood and anxiety. Different factors may be associated with higher levels of mental distress: fear and worry about one’s own health and the health of family members; changes in sleep patterns; worsening of chronic health problems; worsening of pre-existing mental health conditions; and increased use of alcohol, tobacco, or other drugs [17].

Although the pathogenesis, clinical characteristics, and epidemiological impacts of COVID-19 infection have been systematically explored, post-COVID-19 conditions remain poorly understood, with no established etiology, adequate prevention, or definitive treatment [14]. The need for new studies is also highlighted by the high heterogeneity in the available estimates of anxiety and perceived stress and by the methodological variability between investigations [18]. Estimating the burden of anxiety and stress in long COVID can present substantial challenges. These include the length of follow-up, the dynamic nature of the pandemic scenario, the effect of COVID-19 vaccines, and reinfections that contribute to additional risk [9].

Therefore, the prevalence and determinants of anxiety and stress in long COVID are not consolidated in the Brazilian population, and there is a gap in knowledge regarding the predictors for their development. Understanding mental health outcomes is fundamental for implementing comprehensive lines of care and effective, sustainable, and evidence-based public health policies [19].

Therefore, national surveys using a standardized methodology are important for the early identification, monitoring, control, prevention, and treatment of diseases, especially mental health problems during long COVID. In this study, we analyzed the indicators of anxiety and perceived stress in people who have experienced long COVID and determined the predictors associated with its occurrence in the different regional contexts of Brazil.

## 2. Materials and Methods

This cross-sectional study is part of the project “Physical and Psychological Impacts of COVID-19 on the Brazilian Population: Evidence for Technological Interventions in Health”, approved by the Strategic Emergency Program to Prevent and Combat Outbreaks, Endemics, Epidemics, and Pandemics of the Coordination for the Improvement of Higher Education Personnel (CAPES).

This study included 4239 people, of both sexes, aged 18 or over and with at least one diagnosis of COVID-19 proven by laboratory examination, selected for convenience in the five macro-regions that make up the Brazilian territory. The sample estimate was defined based on a sampling plan by geographic stratum, considering the population estimates registered in 2021 in the Department of Informatics of the Unified Health System (DATASUS). In addition, a presumed prevalence of long COVID of 50.2% recorded in the Brazilian population was considered [20].

In Brazil, COVID-19 has affected a representative sample of the general population, making it one of the most affected countries in the world in absolute terms. By December 2024, 39 million cases and 714,000 deaths related to the disease had been confirmed in the country. Its impact has varied between different population segments due to socioeconomic inequalities, housing conditions, and access to health services [21].

Although recruitment took place through social media such as Instagram, WhatsApp, and email, data collection was structured to include participants who did not have technological devices or the skills to use them. In order to make the collection feasible and ensure sample representativeness, data collectors equipped with suitable devices were selected and trained in all regions of Brazil. This method ensured fairness in the collection process, expanding the scope of the study and minimizing technological or socioeconomic barriers that could compromise data collection.

Data collection took place from May 2023 to March 2024. The questionnaires were applied using the Research Electronic Data Capture (REDCAP) platform, version 9.1.0 of 2019, linked to the Ribeirão Preto Nursing School of the University of São Paulo, Ribeirão Preto, Brazil. Two questionnaires were used to assess (1) sociodemographic characteristics and clinical and therapeutic profiles during the acute phase of COVID-19 infection and (2) the presence and persistence of residual symptoms.

Anxiety and perceived stress represent the primary outcomes of this study and were screened using the Brazilian version of the Depression, Anxiety, and Stress Scale (DASS-21). This is a screening instrument made up of twenty-one items that meet the internationally accepted diagnostic criteria for anxiety and stress, which assess the frequency of symptoms perceived by the patient in the previous two weeks [22].

Positive screening for anxiety was measured through the following points: I felt my mouth was dry; I had difficulty breathing at times; I felt shaky; I worried about situations in which I might panic and look ridiculous; I felt I was going to panic; I felt I had no value as a person; I felt afraid for no reason. For stress, the following points were considered: I found it difficult to calm down; I tended to overreact to situations; I felt I was always nervous; I felt agitated; I was intolerant of things that prevented me from continuing what I was doing; I felt I was a bit too emotional/sensitive [13]. The possible answers varied on a Likert scale with four response points: (0) ‘Did not apply at all’, (1) ‘Applied to some degree, or for a short time’, (2) ‘Applied to a considerable degree, or for a good part of the time’, and (3) ’Applied a lot, or most of the time’ [22].

The scale has been validated for Brazil and allows us to identify individuals who are prone to developing anxiety and stress [13,14]. In Brazil, the instrument shows evidence of reliability as estimated by the Cronbach’s alpha coefficient of 0.90 for the stress domain and 0.86 for anxiety. In the literature, it is a valid measure for assessing these outcomes in clinical and non-clinical populations of adults from different cultures, contexts, and ethnicities [14]. It should be noted that anxiety and depression emerged after COVID-19 [22,23].

The GAMLSS class of regression models was used for data processing. This model works with a wide range of distributions, including continuous and discrete variables, asymmetric and/or with heavy tails, which may or may not exhibit heterogeneity in their characteristics. One of the most important stages is defining the distribution of the response variable adopted for the model and the independent variables that will be defined to adjust the data [24]. The study’s outcome variables were the respective total scores for the Anxiety and Stress domains, assessed using the DASS-21 scale.

The covariates included demographic, clinical, and therapeutic information: age (completed years), sex at birth (female, male), marital status (married, single, divorced/separated/widowed), skin color (white/yellow/indigenous, black/brown), education (completed elementary school, high school, college, graduate school), occupation (employed, unemployed/retired, self-employed, student), income in minimum wage (<1, 1, 2, 3, 4, 5, or more), diagnosis before being vaccinated (yes, no), chronic illness (yes, no), severity of illness (mild, moderate, severe), persistence of any physical and/or mental health symptoms for 4 weeks or more (yes, no), seeking health services to investigate/treat these symptoms (yes, no), and hospitalization for any of these symptoms (yes, no).

The variables were selected in two stages. In the first, the presence of multicollinearity between the independent variables was assessed. This assessment determines the entry of variables into the model that are highly correlated with each other. One of the most commonly used measures is the variance inflation factor, whose expression is defined by the following formula:

$${VIF}_{j}=\frac{1}{1-R_{j}^{2}}$$

where $R_{j}^{2}\;$ is the multiple correlation coefficient resulting from the regression of X_j_ on the other p−1 regressors. The greater the degree of dependence of X_j_ on the remaining regressors, the stronger the dependence and the higher the value of $R_{j}^{2}$. A VIF value > 5 was adopted as the cut-off point.

In the second stage, the stepwise variable selection procedure was applied, using the Bayesian Information Criterion for the variables remaining from the first stage [25]. Adjacent models were compared using the likelihood ratio test. In the final model, the corresponding relative increase in average (RA) values derived from the parameters obtained for each of the adjusted models were calculated. All the analyses were carried out using the R program (R Core Team, version 4.3.1, 2023, Ribeirão Preto, Brazil) with a significance level of 5% (α = 0.05) and using the gamlss and mctest packages [26].

This study was approved by the Research Ethics Committee of the Federal University of Paraíba, and a favorable opinion was issued under process number 5.841.147. Participation was voluntary and conditional on signing the online Free and Informed Consent Form. The study also followed the ethical precepts established by Resolution 510/2016 of the National Health Council. One of the guidelines is that it is not compulsory to fill out the research forms in full. This flexibility may have contributed to the occurrence of sample differences between the variables investigated. Despite this, appropriate statistical techniques were used to minimize impacts on the process of analyzing the results.

## 3. Results

This study analyzed data from 4239 individuals who had been diagnosed with COVID-19 by laboratory testing at least once. The majority of participants were female (2987, 70.5%), self-declared brown (2005, 47.4%), single (2544, 60.2%), and living in the north (1170, 34.5%) and northeast (1535, 45.2%). Ages ranged from 18 to 88 years, with a mean age of 32.75 years (SD = 12.80). The predominant income was one minimum wage (1099, 25.9%), 2256 participants (53.3%) had completed high school, and 1950 (46.9%) were active in the labor market.

Although there was a predominance of participants with a single COVID-19 diagnosis, 2700 (65.4%) had recurrent infections, 3659 (89.5%) had mild clinical conditions, and 4030 (98.4%) had high adherence to immunization strategies. Although not predominant in the population investigated, there was a significant indication of chronic diseases (11.8%), including systemic arterial hypertension (58%), diabetes mellitus (32.8%), obesity (33.8%), heart disease (13.2%), cancer (12%), and HIV (6.2%). Of the participants investigated, 1948 (48.5%) had residual symptoms that began between 4 and 12 weeks after the acute phase of COVID-19 infection.

Positive screening for anxiety and perceived stress, measured by the DASS-21, was significantly associated with gender, occupation, income, marital status, schooling, body mass index (BMI), number of COVID-19 diagnoses, severity of clinical condition, presence of comorbidities, and occurrence of prolonged symptoms (*p* < 0.005). There were no differences in positive screening between the self-reported race/skin color variable. The sociodemographic characteristics as well as the clinical conditions during the acute phase of the disease are described in Table 1.

Table 2 shows the results of the regression model adjusted for anxiety in the DASS-21 scale, highlighting the predictors associated with anxiety symptoms in individuals with long COVID. The model included variables such as gender, age, diagnosis of chronic diseases, physical symptoms, need for hospitalization, residual symptoms, and the severity of the infection.

The results indicate that female gender (estimate = 0.2357, *p* < 0.0001), diagnosis of a chronic disease (estimate = 0.1437, *p* = 0.0250), physical symptoms (estimate = 0.2181, *p* = 0.0001), need for hospitalization (estimate = 0.2449, *p* = 0.0401), residual symptoms (estimate = 0.2603, *p* < 0.0001), and moderate or severe infection (estimate = 0.2020, *p* = 0.0054) are significant predictors of increased anxiety symptoms. Age showed a negative relationship with anxiety (estimate = −0.0134, *p* < 0.0001), suggesting that increasing age may be associated with a reduction in anxious symptoms.

Regarding stress, the predictors for occurrence were female gender (estimate = 0.1592, *p* < 0.0001), having a diagnosis of a chronic illness (estimate = 0.1015, *p* = 0.0189), and the presence of physical symptoms (estimate = 0.1313, *p* = 0.0004). The presence of residual symptoms (estimate = 0.1228, *p* = 0.0006) after the acute phase of the infection and the need for home care (estimate = 0.0769, *p* = 0.0407) were also predictors of ongoing stress in long COVID. These results reinforce the importance of clinical and post-infection care factors as key predictors for the development of stress in individuals affected by long COVID (Table 3).

## 4. Discussion

This study analyzed and identified the predictors of positive screening for anxiety and stress among COVID-19 survivors in the Brazilian population. The prevalence ratios were higher among women, people diagnosed with chronic diseases, and those with moderate or severe clinical conditions during the acute phase of the infection. Patients who required hospitalization or home monitoring were also disproportionately affected by anxiety and stress in the post-COVID phase.

Long COVID is a global challenge due to its high prevalence, prolonged duration, and potential for recurrence, as well as its direct impact on the health status and quality of life of this population. Reports on the syndrome are recent, and this study aimed to describe the prevalence and predictors of anxiety and stress after the acute phase of COVID-19 infection in Brazil [27,28].

Although it is still an unknown phenomenon, the recording of anxiety and stress is widely described after severe, moderate, and mild COVID-19 infections [29]. The evidence showed that the temporal follow-up and the development of the symptoms investigated corresponded to the definitions already proposed for long COVID, which considers the presence of repercussions around 4 weeks after the acute phase of the disease, without alternative explanation [30]. A systematic review with 132 studies and 9,320,687 participants reported the global indicators of mental suffering in long COVID. The prevalence of anxiety among patients was estimated at 23% [31].

Although the study showed a predominance of participants with a single diagnosis of COVID-19, recurrent infections were also reported. This demonstrates the vulnerability of part of the population to reinfection, which may be related to factors such as continued exposure, variants of the virus, and insufficient immunity after the first infection [32]. Recurrent infections suggest the need for greater attention to prevention strategies, such as continuous vaccination and reinforcement of sanitary measures, especially in regions with lower vaccination coverage or in more vulnerable socioeconomic groups. It can also have long-term implications, including an increased risk of complications and the development of residual symptoms, which reinforces the importance of monitoring and studying the evolution of reinfection in the population [33,34].

From the same perspective, it was found that the prevalence of mild clinical conditions accounted for the majority of infections. In the literature, the prevalence of people infected with SARS-CoV-2 who developed mild or moderate symptoms without the need for hospitalization is widely reported. According to the World Health Organization (WHO), 80% of COVID-19 cases are mild [29]. Although they do not directly burden the health system with hospitalizations, they still require monitoring measures, especially among vulnerable populations who may develop severe forms of the disease or have prolonged symptoms even after recovery from the acute phase of the disease. Evidence shows that long COVID also has a high prevalence among mild COVID-19 cases [35,36].

When assessing adherence to immunization strategies, the indicators were high. In the context of the pandemic, the high vaccination rate contributed to a significant reduction in serious cases, hospitalizations, and deaths [37]. Despite the political, social, and health crisis that marked immunization strategies in Brazil, the widespread adherence to immunization campaigns demonstrated the population’s confidence in vaccines as an essential public health measure to contain new waves of infection and limit the spread of more aggressive variants of SARS-CoV-2 [38,39].

The clinical characterization also revealed a significant number of chronic diseases, reflecting their high prevalence in the population and their relevance to public health. In Brazil, chronic diseases such as hypertension, diabetes, cardiovascular disease, and obesity affect a significant portion of the population and are responsible for a large proportion of deaths and health complications [40]. These comorbidities compromise the body’s ability to fight infection and increase the possibility of systemic complications, resulting in severe clinical conditions that lead to respiratory failure, the need for mechanical ventilation, higher rates of hospitalization, and death [41].

Among the predictors of anxiety and stress in long COVID, female gender stood out. The greater occurrence of psychosocial alterations among women has been reported in different studies. Although the reasons for this disparity are still emerging, the combination of biological, hormonal, social, cultural, and psychological factors may explain the difference between the sexes.

Women tend to have an exacerbated immune response, which can result in a prolonged inflammatory response and influence women’s greater vulnerability to experiencing long COVID [41,42]. In addition, hormonal fluctuations throughout a woman’s life have a profound impact on mental health. During menstruation, pregnancy, the postpartum period, menopause, and the menstrual cycle, variations in the levels of hormones such as estrogen and progesterone can affect mood, increase vulnerability to stress, and trigger episodes of anxiety [43].

It should also be noted that women face a series of unique burdens that are interrelated and profoundly influence their life experiences. They are often subjected to strict social expectations regarding their behavior, their goals, and how they should balance the multiple roles they play. The traditional role of caregiver, which encompasses responsibility for the family’s well-being, together with the requirement to take on several roles simultaneously can result in continuous emotional exhaustion and a sense of overload. In addition, factors such as greater susceptibility to chronic health conditions, concerns about managing one’s own health, responsibility for running the household and caring for children, the pursuit of quality education and professional success, and a lack of emotional and social support highlight women’s socioeconomic vulnerability and predisposition to developing anxiety and stress [44].

The severity of the acute phase of COVID-19 infection was found to be an important predictor of anxiety, depression, and stress. Although these events were identified in all clinical presentations of the disease, the highest prevalence occurred in more severe patients who required critical care and life-sustaining support. Although still in its infancy, another study aimed to elucidate the influence of inflammatory mechanisms on psychiatric consequences. The results suggest that the disruption of the immune system triggered by infection can induce psychopathology. Associated with this are different psychological stressors represented by fear, social isolation, the psychological impact of a new serious and potentially fatal illness, concerns about infecting others, and stigma [45].

In the same context, clinical comorbidities and obesity increase the risk of mental suffering. These conditions are recognized as risk factors and determinants for more severe clinical outcomes. A higher lethality from COVID-19 is also referenced in this population. Being part of a high-risk group for severe infection may be related to the high prevalence of anxiety, depression, and stress in those with long COVID [46].

Given the complexity of various factors that influence stress and anxiety indicators associated with long COVID, it is essential to adopt an integrated, universal, equitable, and community-centered approach. Strategies that involve health education, financial aid, enhanced social support, labor rights flexibility, nutritional security, and the expansion of mental health services are fundamental in addressing the psychiatric repercussions in COVID-19 survivors [47].

The assessment of long COVID syndrome in Brazil is crucial for understanding the prolonged impact of the pandemic on the country’s public health system. This condition burdens the healthcare system, requires rehabilitation and specialized treatments, and can affect productivity and generate significant socioeconomic impacts. Additionally, the assessment would help to identify inequalities in access to care, particularly among vulnerable populations, and guide more effective public policies, ensuring better resource allocation and the development of treatment protocols adapted to Brazil’s reality. Understanding, preventing, and treating long COVID is critical to mitigate its effects and plan health management strategies in the country.

## 5. Conclusions

Anxiety and stress developed or exacerbated during the post-COVID-19 phase represent significant global challenges. Our study, conducted in Brazil (2023–2024), identified that women and people with chronic diseases, moderate or severe clinical conditions in the acute phase of the infection, and the need for hospitalization are disproportionately affected due to various socioeconomic and biological factors. Although the underlying pathophysiology remains unclear, targeted interventions can provide relief and improve overall well-being. Knowing these repercussions can direct the planning of mental health care and the structuring of effective, sustainable, and evidence-based public policies.

## Figures and Tables

**Table 1:** Sociodemographic, clinical, and therapeutic characteristics by prevalence of positive screening for anxiety and stress in patients who experienced long COVID in Brazil.

Variables	N	Mean	SD	Anxiety			Stress		
				Mean	SD	*p*-Value	Mean	SD	*p*-Value
Sex									
Male	2987			2.94	3.85	0.0000	4.57	4.80	0.0000
Female	1251			4.25	4.46		6.47	5.28	
Age									
Occupation		35.75	12.80						
Employee	1443			3.43	4.20	0.0000	5.34	5.03	0.0000
Unemployed/retired	385			3.85	4.37		5.28	5.20	
Income									
>1 Minimum wage (SM)	675			4.51	4.47	0.0000	6.69	5.32	0.0000
1 SM	792			4.11	4.54		5.75	5.23	
Race									
White/yellow/indigenous	1410			3.85	4.31	0.9531	6.13	5.19	0.0219
Black	296			3.79	4.24		5.77	5.22	
Marital status									
Married or living as married	1017			3.15	3.98	0.0000	4.91	4.85	0.0000
Single	1949			4.27	4.44		6.49	5.32	
Education									
Completed higher education (Graduation)	679			3.66	4.14	0.0000	5.59	5.10	0.0000
<Completed elementary school	164			2.96	4.14		4.04	4.92	
CIM									
Appropriate weight	1325			3.83	4.23	0.0025	6.13	5.29	0.0017
Low weight	111			4.78	4.82		6.99	5.45	
Number of COVID-19 diagnoses									
1	2072			3.66	4.23	0.0001	5.65	5.18	0.0001
2	850			4.00	4.23		6.19	5.15	
Clinical picture									
Level	2823			3.72	4.24	0.0000	5.84	5.22	0.0134
Moderate/severe/critical	317			5.15	4.87		6.48	5.18	
Specialized service									
Basic care	642			3.87	4.33	0.0259	5.80	5.30	0.0112
Hospital care	753			4.18	4.46		6.37	5.27	
Diagnosis of COVID-19 before being vaccinated									
No	1279			3.58	4.17	0.0040	5.76	5.14	0.2271
Yes	1792			4.05	4.44		6.01	5.27	
Chronic illnesses									
No	2623			3.68	4.28	0.0000	5.70	5.15	0.0000
Yes	391			4.58	4.35		6.83	5.41	
Physical symptoms									
No	1375			2.78	3.67	0.0000	4.56	4.67	0.0000
Yes	1772			4.68	4.61		6.92	5.38	
Hospital stay									
No	3036			3.78	4.26	0.0000	5.85	5.16	0.0504
Yes	105			5.98	5.38		7.25	6.30	
Prolonged symptoms after the acute phase of infection									
No	1638			2.98	3.76	0.0000	4.89	4.81	0.0000
Yes	1510			4.78	4.67		6.96	5.41	

**Table 2:** Predictors for anxiety after long COVID.

Category	Estimate	*p*-Value
Female	0.2357	0.0000
Age	−0.0134	0.0000
Diagnosis of chronic diseases	0.1437	0.0250
Physical symptoms	0.2181	0.0001
Hospitalization	0.2449	0.0401
Residual symptoms	0.2603	0.0000
Moderate or severe infection	0.2020	0.0054

**Table 3:** Predictors for stress after long COVID.

Category	Estimate	*p*-Value
Female	0.1592	0.0000
Age	−0.0091	0.0000
Chronic illness	0.1015	0.0189
Physical symptoms	0.1313	0.0004
Residual symptoms	0.1228	0.0006
Home care	0.0769	0.0407

## Data Availability

Data are available on request due to restrictions. The data presented in this study are available on request from the corresponding author.
